# The Treatment of Fractures from a Common-Sense Point of View

**Published:** 1907-08-24

**Authors:** 


### Points in Surgery.

### THE TREATMENT OF FRACTURES FROM A COMMON-SENSE POINT OF VIEW.

### II. SPLINTS AND RETENTIVE APPARATUS.

When the ends of a fractured bone have been got into apposition, the surgeon must direct his attention to keeping them at rest in that position until union has occurred. This is done with some form of retentive apparatus. Nothing strikes the junior house surgeon with such force as the difference between the simplicity of the forms of retentive apparatus in actual use and the complicated nature of the various splints whose merits are advocated in the text-books of surgery. Intricate mechanical appliances like the double inclined plane and the Middeldorf triangle are often described in full, but are rarely, if ever, seen in the wards.

The wooden splint is the simplest possible form of retentive apparatus. Its object is not only to keep the fragments in position, but to immobilise the limb. Movement at the joints both above and below the fracture should be prevented. For example, in fractures of the middle of the shaft of the humerus it is important to keep both the elbow and shoulder at rest. The former object is achieved by using an angular splint (the internal angular is the easiest to apply and the most comfortable, and is the one in general use), and the latter by strapping or bandaging the whole arm to the side. Neglect of these precautions almost always leads to non-union or to pseud-arthrosis, either of which complications may prolong the patient's convalescence for an indefinite period.

The splint must be well padded to avoid pressure over bony prominences, and must be firmly applied. The exact amount of pressure required in bandaging a splint to a limb can only be obtained by experience. It is obvious that if it is applied too lightly it will not keep the ends in apposition, while if the bandage is too tightly put on it causes the patient great pain, and may lead to œdema of the limb, or even to actual gangrene. It must be remembered that some effusion will surely take place later, and that a bandage which is just firm enough when the splint is applied may become dangerously tight within the next twenty-four hours. It is, therefore, a safe rule to examine all cases of fracture on the next day, particularly with a view to ascertaining whether there is any œdema of the extremities of the limb. If any is present the bandage must be reapplied, but if the patient does not complain of pain there is no reason to disturb it.

There is hardly any form of fracture occurring in a limb which cannot be efficiently treated by means of suitable wooden splints but there is one form of splint which is of great service in treating fractures of the tibia and fibula, the Bavarian plaster-of-Paris splint. It is very comfortable, and can be readily adjusted. It is a modification of Croft's splint, and has this advantage over the latter that it is more easy to adjust, and does not demand a series of exact measurements. It is made as follows: Two pieces of house-flannel of sufficient size to enclose the limb are stitched together longitudinally down their centre. The limb is placed on these, and the inner piece of flannel is stitched over the front of the limb down the middle line of the leg and along the middle of the sole of the foot, so that it fits it closely like a stocking, and the flannel is then cut round about two inches from the stitches so as to leave a projecting fringe. The outer piece of flannel is now cut to shape the limb exactly, so that its edge corresponds to the line of stitches in the first piece. The interval between the two pieces of flannel is then filled with plaster of Paris, and the projecting fringes of the inner piece of flannel are turned down over the outer piece; a thin coating of plaster may be applied over all with the hands. The splint should extend from above the knee to a transverse line round the fifth metatarsophalangeal joint. When the plaster has set the splint can be easily opened down the front of the leg and the sole of the foot, because the projecting pieces of the inner layer of flannel protects the stitches from being embedded in the plaster, and it can be removed from the limb because there is no plaster in the original line of suture between the two pieces of flannel behind: thus a natural hinge is formed, so that the splint can be taken off without cracking the plaster. This form of splint will be found most simple and most efficient.

Cases, however, constantly occur in which the ordinary forms of retentive apparatus, however skilfully applied, are incapable of keeping the fragments in apposition. For instance, in fractures of the femur, which will be dealt with in detail in a subsequent article, it is a routine practice to apply extension to the limb for that reason. In these cases the surgeon has the choice between applying some form of extension apparatus and fixing the fragments by an open operation. Wherever the former alternative is possible there should be no hesitation in adopting it, since there are many objections to open operation. Some well-known surgeons advocate wiring the bones together in nearly all forms of simple fracture. But it must be remembered that by open operation a simple fracture is at once converted into a compound one. This is no great matter if asepsis can be assured; but if not the ultimate result will be worse than if no operation had been undertaken. What, then, are the indications for wiring the fragments by open operation in simple fractures? It is justified in the following conditions: (i.) Where there is wide separation of the fragments, as in fracture of the patella or olecranon; (ii.) in very oblique fractures of a long bone where the fragments override and much deformity is likely to result; (iii.) where the fragments cannot be retained in position by the ordinary forms of apparatus. A good example of this is afforded by supracondyloid fractures of the humerus in muscular men. The lower fragment is pulled upwards by the triceps. The ordinary forms of splint are incapable of retaining the fragments in apposition, and it is impossible to apply satisfactory extension in this situation.

